# Supplementary material for: Endogenous labour flow networks
Source: EPJ Data Sci. 2025 May 21;14(1):39. doi: 10.1140/epjds/s13688-025-00539-9 (PMC12095427; doi:10.1140/epjds/s13688-025-00539-9)
Supplement: Supplementary file 1 — (PDF 11.7 MB) [file 13688_2025_539_MOESM1_ESM.pdf]

Supplementary Information: Endogenous Labour Flow Networks

Kathyrn R. Fair[1,*] and Omar A. Guerrero[1,2]

[1]The Alan Turing Institute, London

[2]Centro de Estudios Espinosa Yglesias, Mexico City, Mexico

[*]kfair@turing.ac.uk

Defining a steady state

Let $\mathbb{R}_t$, $\mathbb{I}_t$, and $\mathbb{O}_t$ represent the model's regional, and occupational transition density matrices for the corresponding LFNs in period t. The corresponding matrices for the observed LFNs are defined by $\mathcal{R}$, $\mathcal{I}$, and $\mathcal{O}$. Thus, the error function for period t, given by Ξ_t, is defined as

$$\Xi_t = \frac{1}{3}\left(\|\mathbb{R}_t - \mathcal{R}\|_F + \|\mathbb{I}_t - \mathcal{I}\|_F + \|\mathbb{O}_t - \mathcal{O}\|_F\right), \tag{1}$$

where $\|\cdot\|_F$ denotes the Frobenius norm of a matrix.

The steady state is reached if the following condition holds:

$$\left| \left(\frac{1}{k} \sum_{t=T-k}^{T} \Xi_t \right) - \left(\frac{1}{k} \sum_{t=T-k-l}^{T-l} \Xi_t \right) \right| < \epsilon \tag{2}$$

where k is the size of the smoothing window, l is the lag, T is the current timestep of model, and ϵ is a small, positive threshold parameter. In other words, the steady state is detected if there is stability and convergence in terms of the labour flows of the model.

Community detection

The goodness of fit obtained from our calibration procedure across the three types of LFNs can also be evaluated using the Variation of Information (VOI) for communities found in the LFNs. Community detection is performed using two algorithms suitable for weighted directed networks; RB pots [1, 2] and Surprise Communities [3]. These algorithms were chosen as they require minimal hyperparameter tuning. However, we emphasise that the insight that can be gained from performing community detection on networks with so few nodes (resp. 21, 21, and 9 for the region, industry, and occupation LFNs) is limited. We provide a comparison of the VOI between the observed and simulated LFNs, and between the observed LFN and a rewired version of the observed LFN. Rewiring is performed via strength-preserving network randomization using simulated annealing [4–6].

For both the region and industry LFNs, the VOI between the communities identified in the observed and simulated LFNs is lower than the VOI for the

communities identified in the observed and rewired LFNs, for both community detection methods. A larger VOI value indicates greater distance between two computed network clusterings. This demonstrates that the simulated LFNs capture the community structure of the observed LFNs to a greater extent than would be expected by chance. For the occupation LFN, the VOI is higher between the observed and simulated LFNs than between the observed and rewired LFNs, for both community detection methods. However, given that this network contains only 9 nodes, it is difficult to draw any meaningful conclusions from this result.

Network	VOI (RB pots)	VOI (Surprise Communities)
Region	0.19 (2.66)	0.00 (1.14)
Industry	1.35 (3.05)	0.00 (0.80)
Occupation	0.22 (0.00)	0.44 (0.00)

Table S1: **Comparison of variation of information (VOI) between communities for observed, simulated, and rewired LFNs.** Each of the VOI columns presents results using a different community detection algorithm (RB pots and Surprise Communities). Values presented first in each cell correspond to calculations comparing observed LFNs to those generated from Monte Carlo simulations, while values subsequently presented in brackets correspond to calculations performed to compare observed LFNs with their associated rewired LFN.

Model validation

To validate the model, we examine the outputs of simulation in the presence of shocks under two scenarios: (1) a networked system, where similarities

between industries, occupations, and regions influence the matching of individuals with vacant positions as well as the hiring process and (2) a well-mixed system where individuals are randomly matched with and hired to vacant positions, as is implicitly assumed in aggregate matching (job search) functions [7]. For simplicity, we focus on a shock that affects the characteristics of the job positions within the Public sector, as discussed in Results - Shocks in the main text. The time it takes the system to reach a steady state, the behaviour of the overall unemployment rate, and of the inflows and outflows to the Public sector are examined, as we can place these results in the context of previous work modelling LFNs.

First we observe that the LFN takes longer to stabilise both after model initialisation, and after the introduction of a shock (i.e. the time-series, which terminate when the simulation has reached a steady-state post-shock, are longer for the networked system than for the well-mixed system) (Figure S1 and Figure S2). We observed this behaviour in a previous study performed using a stochastic process describing firm-level LFNs. There we introduced a shock that reduced the hiring rate of firms, and showed that the unemployment rate took significantly longer to stabilise in the aftermath of this shock when the system is networked (i.e. incorporates LFN topology) [8]. We also have previously shown, in another study of firm-level LFNs using stochastic processes, that a networked system should lead to a higher aggregate unemployment rate [9]. This difference is also one we observe here (Figure S1) , both pre- and post-shock. These results are expected; incorporating network structure means we are accounting for labour market frictions that inhibit individuals

from moving freely from one position to another, and thus we should observe a higher aggregate unemployment rate.

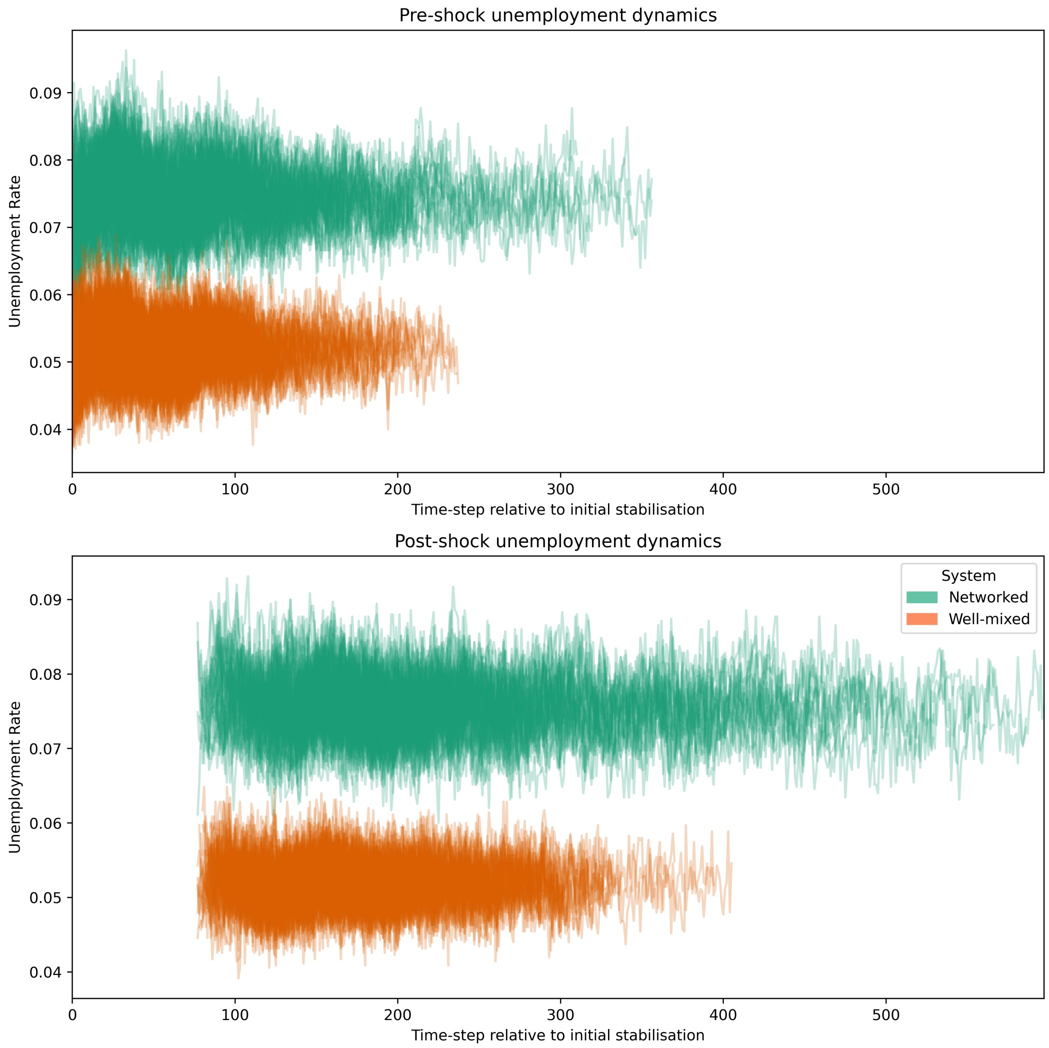

Figure S1: **Impact of system structure on unemployment.** Subplots indicate pre- and post-shock aggregate unemployment dynamics. Each line corresponds to a model realisation from a suite of Monte Carlo simulations performed with $N = 3500$.

As discussed in the Introduction of the main text, previous modelling approaches have assumed an exogenous LFN structure. Thus, these approaches

do not provide any insight into the qualitative changes to flows that result from a shock. In contrast, our endogenous generation of the LFN allows us to observe such changes (Figure S2 shown in green). A shock to the public sector resulting in a decrease in inflows, and a slightly smaller decrease in outflows, leading to a negative net inflow to this sector. While the shock we apply is highly stylised, this result shows the potential of the model to gain highly granular insights on the impacts of shocks on the labour market. We also show that these results would not be evident from a well-mixed model (Figure S2 shown in orange), where the flows to/from the shocked sector are insensitive to this shock.

The reader may want to see validation of the model against an empirical exogenous shock (e.g. the COVID-19 pandemic). However, there are several factors that render a comparison impossible. Previous approaches to studying such shocks do not examine labour flows, thus there are no appropriate comparisons for our model outputs within the existing literature. Additionally, our model is calibrated in the steady state, thus we would require a post-COVID steady state to have been reached to accurately judge model performance. Given the short period of time since the pandemic, and the ongoing fallout (along with the contamination of these data with Brexit impacts), it is unlikely that we have reached a new labour market steady state to which we could compare. Additionally, data availability issues would make such an analysis impossible at this time. To construct our labour flow networks (LFNs), we had to aggregate a decade of job-to-job movements within the UK labour market to obtain enough flows. Even if we made the questionable assumption that

the COVID-19 shock only impacted the labour market in 2020-21, we would be left with less than 2 years of post-COVID data to work with, as the latest release of the UK Labour Force Survey dataset used is from June 2023. This would result in an extremely sparse observed LFN, rendering any calibration ineffective, as there would not have been enough job movements to accurately capture the UK LFN. Finally, and perhaps most crucially, at the time of writing the ONS has suspended release of LFS data from August 2023 onwards, as falling survey response rates have made these data unreliable [10]. While they are introducing major changes to address these issues, the unreliability of the currently available data means that a COVID-19 shock scenario is not possible with these data. However, these sorts of analyses could be performed in future, should better data become available. This is something we are working towards but falls beyond the scope of this paper's goal – to provide a theoretical proof of concept for this type of model.

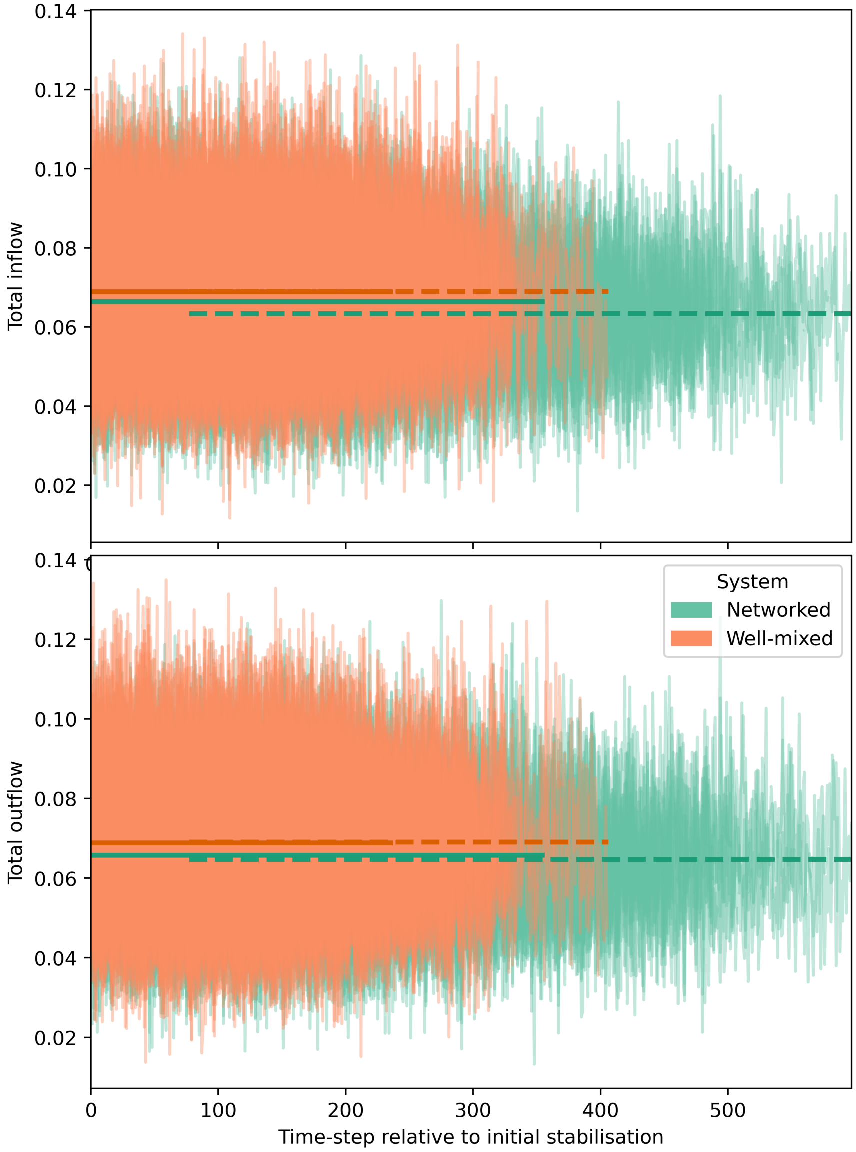

Figure S2: **Impact of system structure on labour flows.** Subplots indicate flows for the public sector. Each time-series line corresponds to a realisation from a suite of Monte Carlo simulations performed with $N = 3500$. Solid horizontal lines indicate the mean y-axis value over the span of time-steps during which simulations were running in the absence of a shock (i.e. pre-shock steady state), dashed lines indicate the mean y-axis value over the time-span during which the simulations ran post shock (until their post-shock steady state was achieved). Time-steps taken to achieve stability vary between model realisations, meaning the time at which the shock is introduced will also vary, hence the overlap in the time periods covered by these lines.

Dictionaries

Geographical Region	Label
Tyne and Wear	Tyne & Wear
Rest of North East	R. of North East
Greater Manchester	G. Manchester
Merseyside	Merseyside
Rest of North West	R. of North West
South Yorkshire	South Yorkshire
West Yorkshire	West Yorkshire
Rest of Yorkshire and Humberside	R. of Yorks. and H.
East Midlands	East Midlands
West Midlands (metropolitan county)	W. Midlands (MC)
Rest of West Midlands	R. of W. Midlands
East of England	East of England
Central London	Central London
Inner London	Inner London
Outer London	Outer London
South East	South East
South West	South West
Wales	Wales
Strathclyde	Strathclyde
Rest of Scotland	R. of Scotland
Northern Ireland	N. Ireland

Table S2: **Region dictionary.** Labels used to refer to geographical regions within the UK throughout text.

Industry section	Label
Agriculture, forestry and fishing	Agriculture
Mining and quarrying	Mining
Manufacturing	Manufacturing
Electricity, gas, steam and air conditioning supply	Electricity
Water supply, sewerage, waste management and remediation activities	Water
Construction	Construction
Wholesale and retail trade, repair of motor vehicles and motorcycles	Motor trade
Transportation and storage	Transport
Accommodation and food service activities	Accommodation
Information and communication	Infocomm
Financial and insurance activities	Finance
Real estate activities	Real estate
Professional, scientific and technical activities	Science & tech
Administrative and support service activities	Admin
Public administration and defence, compulsory social security	Public
Education	Education
Human health and social work activities	Health & social
Arts, entertainment and recreation	Entertainment
Other service activities	Other service
Activities of households as employers; undifferentiated goods- and services-producing activities of households for own use	Household
Activities of extraterritorial organisations and bodies	Extra orgs

Table S3: **Industry dictionary.** Labels used to refer to SIC industry sections throughout text.

Occupation major group	Label
Managers, directors and senior officials	Manager
Professional occupations	Professional
Associate professional occupations	Ass. prof.
Administrative and secretarial occupations	Admin
Skilled trades occupations	Trades
Caring, leisure and other service occupations	Leisure
Sales and customer service occupations	Sales
Process, plant and machine operatives	Machine op.
Elementary occupations	Elementary

Table S4: **Occupation dictionary.** Labels used to refer to SOC major groups throughout text.

Supplementary figures

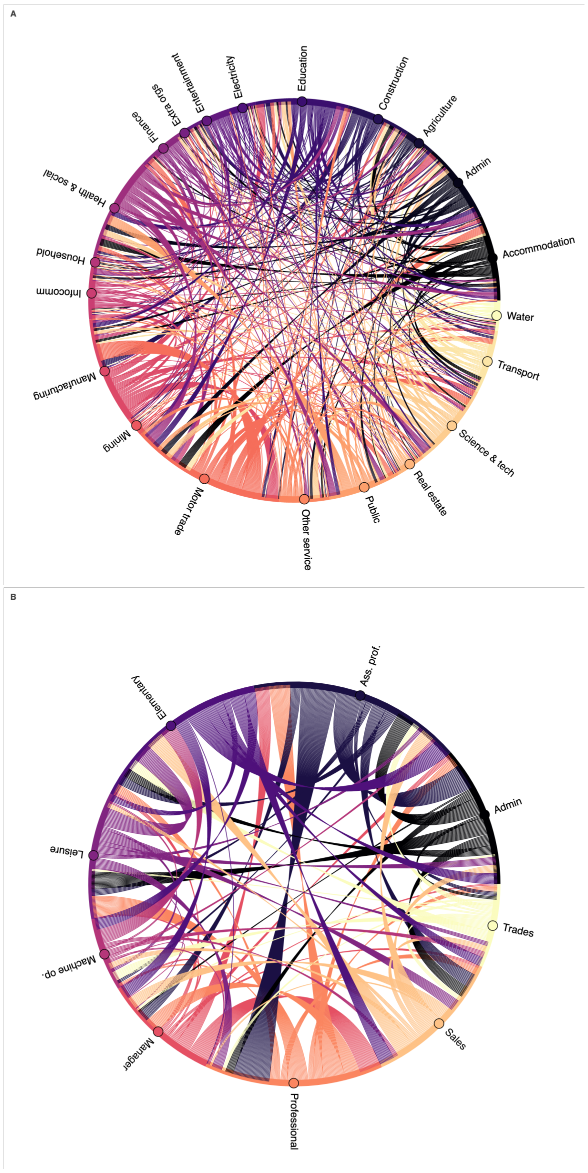

Figure S3: **Simulated labour flows within the UK.** Subplots indicate inter-group labour flows between a) industries and b) occupations. Only inter-group flows are displayed, as intra-group flows tend to be substantially higher due to the localised nature of job search. The colour of a flow corresponds to the group where that flow originated. Labels and descriptions for industries and occupations are provided in Table S3, and Table S4.

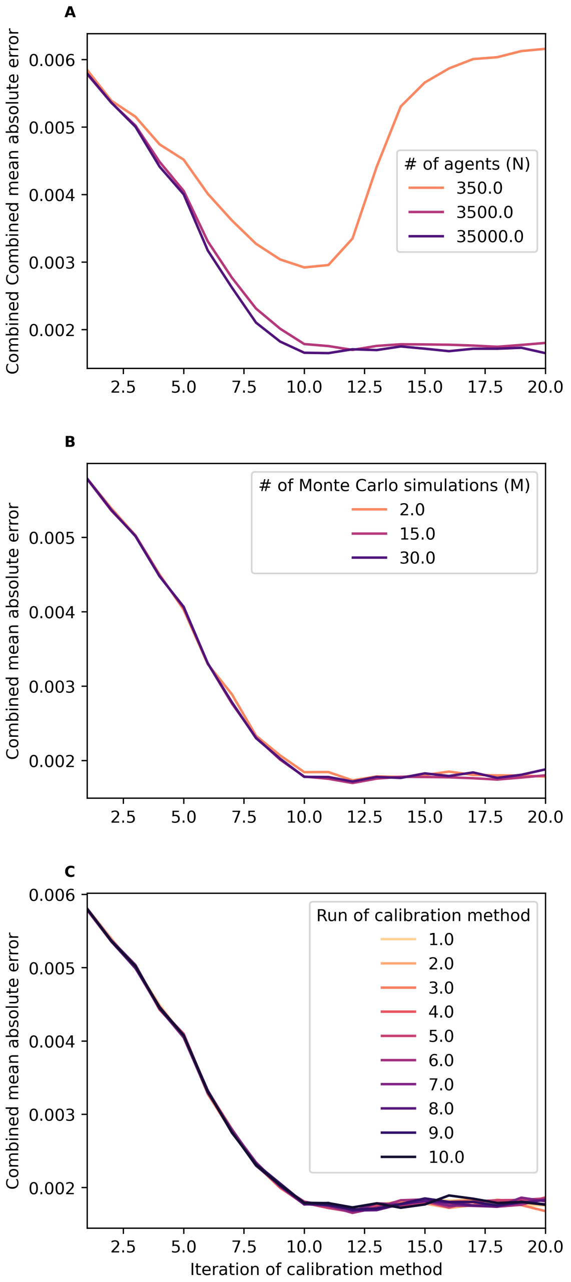

Figure S4: **Robustness of chosen calibration method.** The calibration method is robust to changes in a) the number of agents N (except in the case of very small N) and b) the number of Monte Carlo simulations. Results also hold across multiple runs of the calibration method (with $N = 3500$), as shown in c).

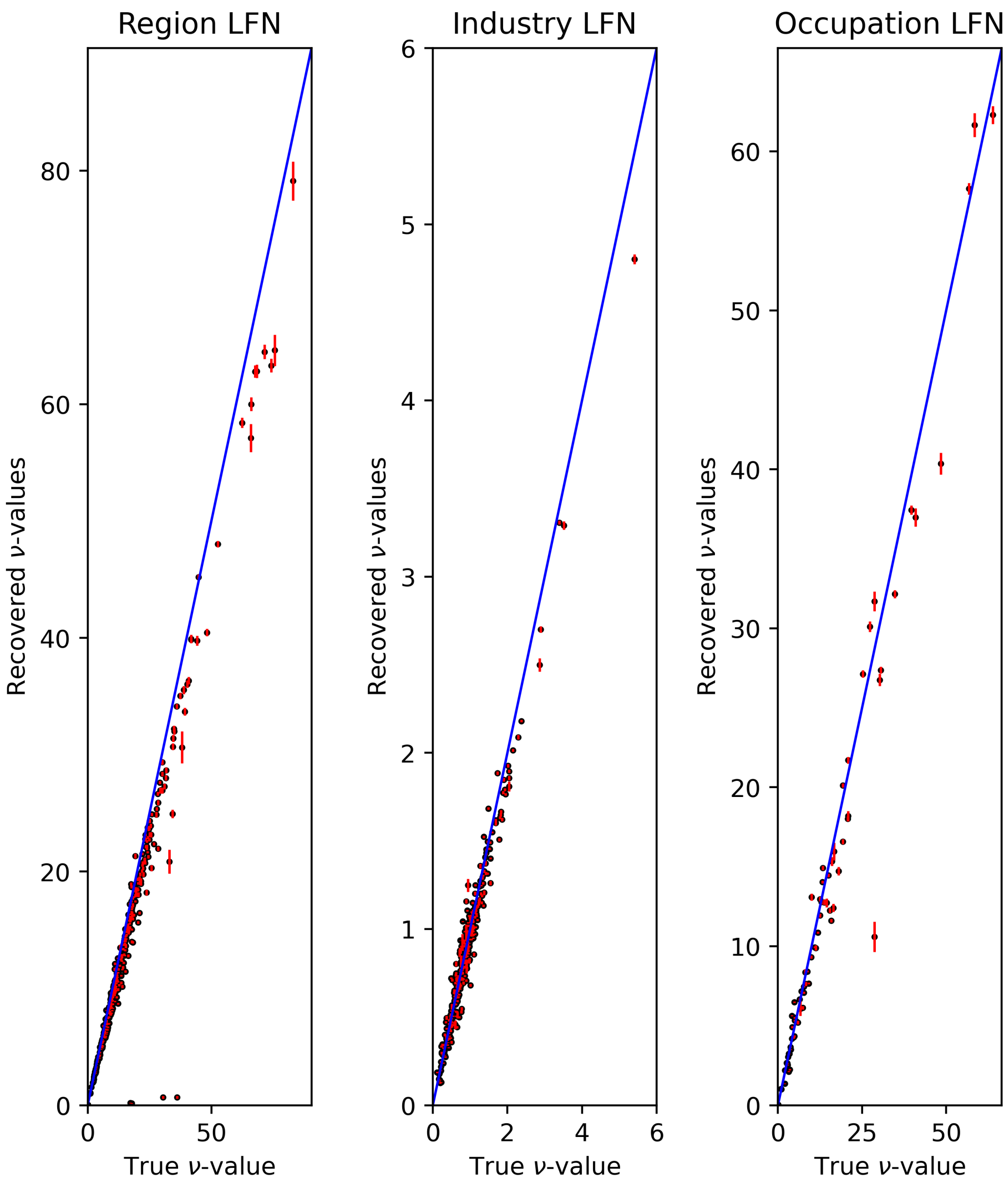

Figure S5: **Results of parameter recovery exercise.** For each ν-value, we make 100 attempts to recover the true value by running the calibration algorithm with the objective of fitting to the LFNs generated by a simulation using the true ν-values. For each set of 100 recovered ν-values the mean is indicated by a black point, and the 99% confidence interval is indicated by a red line. The $y = x$ line is indicated in blue. For each calibration run a suite of Monte Carlo simulations was performed with $N = 3500$.

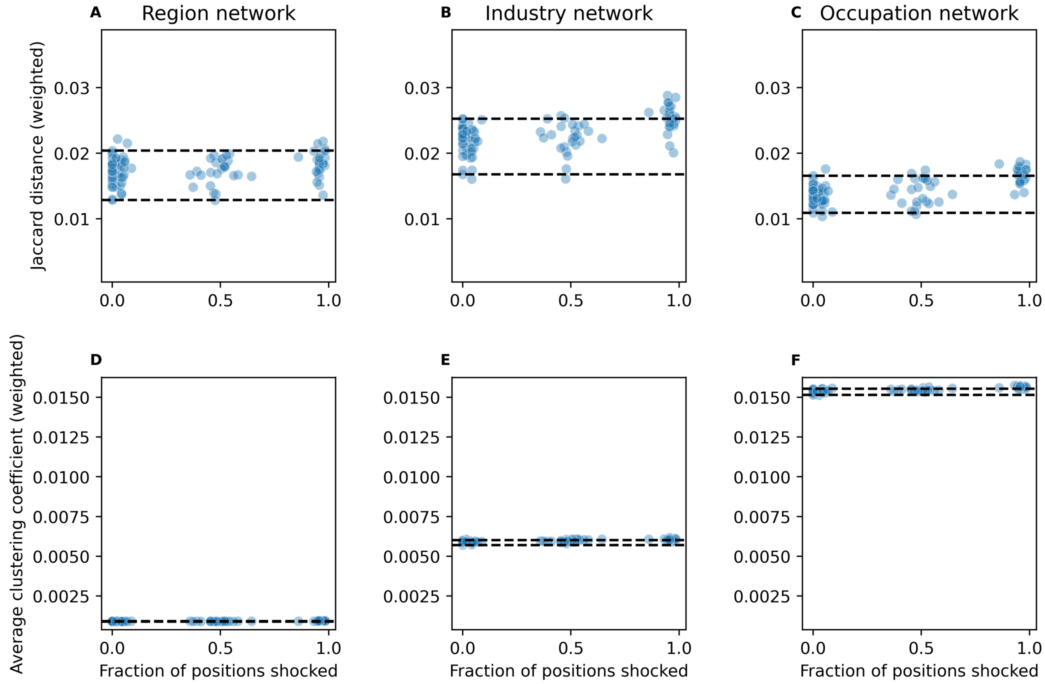

Figure S6: **Relationship between the size of a shock that decreases average wages and the impact of that shock on labour flows.** Subplots indicate weighted (a-c) Jaccard distance and (d-f) average clustering coefficient values for the region, industry, and occupation LFNs. Each point corresponds to the average value taken across a suite of Monte Carlo simulations using the same set of shocked industries. Dashed lines indicate the range of values obtained from simulations where no shock has occurred. This variation between simulations in the absence of shocks is a result of the stochastic nature of the model.

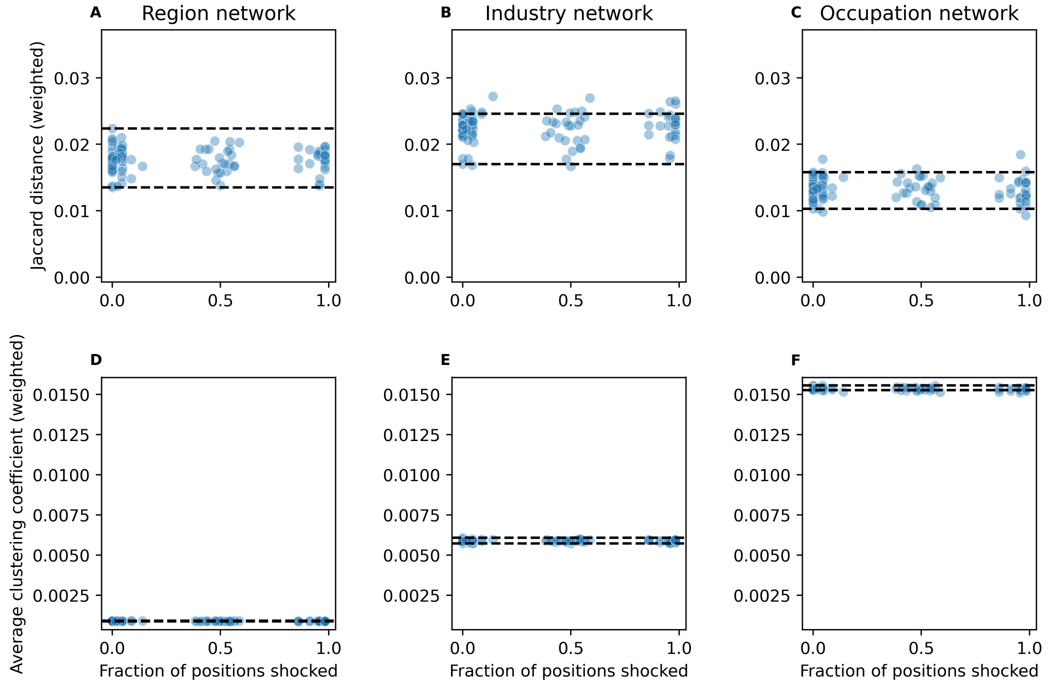

Figure S7: **Relationship between the size of a shock that increases average wages and the impact of that shock on labour flows.** Subplots indicate weighted (a-c) Jaccard distance and (d-f) average clustering coefficient values for the region, industry, and occupation LFNs. Each point corresponds to the average value taken across a suite of Monte Carlo simulations using the same set of shocked industries. Dashed lines indicate the range of values obtained from simulations where no shock has occurred. This variation between simulations in the absence of shocks is a result of the stochastic nature of the model.

References

[1] Jörg Reichardt and Stefan Bornholdt. Statistical mechanics of community detection. *Physical Review E—Statistical, Nonlinear, and Soft Matter Physics*, 74(1):016110, 2006.

[2] Elizabeth A Leicht and Mark EJ Newman. Community structure in directed networks. *Physical review letters*, 100(11):118703, 2008.

[3] Vincent A Traag, Rodrigo Aldecoa, and J-C Delvenne. Detecting communities using asymptotical surprise. *Physical review e*, 92(2):022816, 2015.

[4] Bratislav Mišić, Richard F Betzel, Azadeh Nematzadeh, Joaquin Goni, Alessandra Griffa, Patric Hagmann, Alessandro Flammini, Yong-Yeol Ahn, and Olaf Sporns. Cooperative and competitive spreading dynamics on the human connectome. *Neuron*, 86(6):1518–1529, 2015.

[5] Mikail Rubinov. Constraints and spandrels of interareal connectomes. *Nature communications*, 7(1):13812, 2016.

[6] Filip Milisav, Vincent Bazinet, Richard Betzel, and Bratislav Misic. A simulated annealing algorithm for randomizing weighted networks. *bioRxiv*, pages 2024–02, 2024.

[7] Christopher A. Pissarides. *Equilibrium Unemployment Theory, 2nd Edition*, volume 1. The MIT Press, 2000.

[8] Omar A. Guerrero and Eduardo Lopez. Understanding Unemployment in the Era of Big Data: Policy Informed by Data-Driven Theory. SSRN Scholarly Paper 2716264, Social Science Research Network, Rochester, NY, January 2016.

[9] Robert L. Axtell, Omar A. Guerrero, and Eduardo López. Frictional unemployment on labor flow networks. *Journal of Economic Behavior & Organization*, 160:184–201, April 2019. ISSN 0167-2681. doi: 10.1016/j.jebo.2019.02.028.

[10] Office for National Statistics. Labour force survey: planned improvements and its reintroduction. `https://www.ons.gov.uk/employmentandlabourmarket/peopleinwork/employmentandemployeetypes/methodologies/labourforcesurveyplannedimprovementsanditsreintroduction`, 2023.
